# Effect of *Agaricus bisporus* Polysaccharides on Human Gut Microbiota during *In Vitro* Fermentation: An Integrative Analysis of Microbiome and Metabolome

**DOI:** 10.3390/foods12040859

**Published:** 2023-02-17

**Authors:** Hui Duan, Qun Yu, Yang Ni, Jinwei Li, Liuping Fan

**Affiliations:** 1 State Key Laboratory of Food Science and Technology, Jiangnan University, Wuxi 214122, China; 2 School of Food Science and Technology, Jiangnan University, Wuxi 214122, China; 3 National Engineering Research Center for Functional Food, Jiangnan University, Wuxi 214122, China

**Keywords:** *Agaricus bisporus*, polysaccharides, gut microbiota, metabolome, *Bacteroides*, *Bifidobacterium longum*

## Abstract

*Agaricus bisporus* polysaccharide (ABP) is an important active component in edible mushrooms, but its interaction with gut microbiota is unclear. Therefore, this study evaluated the effect of ABP on the composition and metabolites of human gut microbiota by *in vitro* batch fermentation. The main degrading bacteria for ABP were *Bacteroides*, *Streptococcus*, *Enterococcus*, *Paraprevotella, Bifidobacterium*, *Lactococcus*, *Megamonas*, and *Eubacterium*, whose relative abundances increased during 24 h of *in vitro* fermentation. The short-chain fatty acids (SCFAs) content also increased more than 15-fold, accordingly. Moreover, the effects of ABP on the relative abundance of *Bacteroides* (*Ba.*) and *Bifidobacterium* (*Bi.*) at the species level were further determined. ABP can enrich *Ba. thetaiotaomicron*, *Ba. intestinalis*, *Ba. uniformis*, and *Bi. longum*. PICRUSt analysis revealed that the catabolism of ABP was accompanied by changes in the metabolism of carbohydrates, nucleotides, lipids and amino acids, which were also supported by metabonomic results. It is worth mentioning that, after 24 h fermentation, the relative amounts of gamma-aminobutyric acid (GABA), nicotinamide and nicotinamide adenine dinucleotide (NAD^+^) had 14.43-, 11.34- and 15.36-fold increases, respectively, which were positively related to *Bacteroides* (*Ba. thetaiotaomicron*, *Ba. intestinalis*), *Streptococcus*, and *Bi. longum* (|r| > 0.98). These results laid the research foundation for exploring ABP as a potential prebiotic or dietary supplement for the targeted regulation of gut microbiota or metabolites.

## 1. Introduction

The human gut microbiota, as a diverse ecosystem and “superorganism”, has attracted increasing attention due to its superb metabolic capacity and vital roles in human health and physiology [1,2]. Accordingly, there is growing interest in how to manipulate gut microbiota [3]. The global market targeting gut microbiota therapies and diagnostics was estimated at approximately USD 2.8–4.0 billion in 2019 and is expected to more than triple to USD 7.5–15.0 billion by 2024 [3,4]. 

Undoubtedly, complex polysaccharides are a major driving force in shaping the human gut microbiota. They encode abundant carbohydrate-active enzymes (CAZymes) and thereby break down polysaccharides into monosaccharides, which are further fermented into short-chain fatty acids (SCFAs) in the cecum and colon [5]. SCFAs, including acetate, propionate, and butyrate, are important energy and signaling molecules, thus affecting various physiological processes. For example, acetate can relieve colonic inflammation in mice by stimulating a G-protein-coupled receptor 43 [6]. In addition to acting on the gastrointestinal tract, the acetate can enter the circulation and then directly influence the adipose tissue, brain, and liver, inducing overall beneficial metabolic effects [7].

*Agaricus bisporus* is an important edible and medicinal mushroom, and its active polysaccharide (ABP) can regulate gut microbiota and promote gut health [8,9,10]. Recently, health-promoting functions of ABP including anti-aging and immunoregulation have been reported. For example, ABP had potential anti-aging effects on the liver, kidney, brain, and skin in d-galactose-induced aging mice, and the mechanism may involve enhancing the antioxidant status and improving the lipid metabolism [11,12]. Oral microcapsules loaded with ABP can enhance NK cell cytotoxic effects against colon cancer cells, which may be used for natural killer cells mediated colon cancer immunotherapy [13]. 

The beneficial effects of mushroom polysaccharides may be mediated by gut microbiota, including increases in beneficial bacteria and SCFAs production and the inhibition of pathogenic bacteria [14,15]. For example, the modulatory effects of various mushroom polysaccharides on the intestinal immune response were related to the improvements of the gut microbial communities in aquatic species [15]. However, the interaction between ABP and gut microbiota remains unclear. *In vitro* batch fermentation has been widely used to research gut microbiota fermentation of different polysaccharides, exploring their effects on community structure, their degradation products, and their function, which is helpful to promote the development of personalized nutrition strategies [16]. Therefore, we evaluated the interaction between ABP and gut microbiota, especially the effect of ABP on the composition and metabolome of human gut microbiota, which laid the research foundation for exploring ABP as a potential prebiotic and its precision nutrition strategies.

## 2. Materials and Methods

### 2.1. Preparation of A. bisporus Polysaccharides 

ABP was extracted from dried *A. bisporus* powders (60 mush) with a 1:20 solid–liquid ratio (deionized water) at 100 °C for 1.5 h (extract twice). The two extracted solutions were combined and concentrated at 8000× *g* for 10 min. The supernatant was concentrated to 1/10 of the original volume with a rotary evaporator (IKA HB 10, Staufen, Germany). After 75% of alcohol precipitated, Sevag deproteinized, dialyzed and freeze-dried (CHRIST Alpha 1–4 LSCbasic, Ostrode, Germany), the crude ABP powder was obtained to be used in the following analysis [17].

### 2.2. Preparation of Fecal Samples

Five healthy adult volunteers (age 20–30 years with BMI 18.5–24, 2 males and 3 females) were recruited at Jiangnan University, Wuxu, China. All volunteers (without intestinal diseases) had not received antibiotics or probiotic products for at least 3 months. The study was approved by the Ethics Committee of Jiangnan University (JNU20220901IRB007). The 5.0 g fresh feces were collected and homogenized (2 min) with 35 mL PBS (pH = 7.4, 0.1% cysteine). After centrifugation (600× *g*, 10 min), the supernatant was placed into an anaerobic workstation immediately for the following batch culture fermentation [18].

### 2.3. In Vitro Batch Culture Fermentation

The gut microbiota medium (GMM) with minor modifications was used for *in vitro* batch fermentation [19]. Specifically, the carbon source and SCFAs were removed, and 5 g/L ABP was added as the sole carbon source in the culture medium. The fermentation process was conducted according to a previous study [18]. The 5 mL of human fecal suspension was added to 45 mL of GMM medium, and then incubated at 37 °C in the anaerobic workstation. The samples were collected at 0, 6, 12, 18, 24, 48, and 96 h, respectively, to determine the pH value and the total bacteria load (OD_600_). After centrifugation (8000× *g*, 15 min), the supernatants and precipitation were stored at −80 °C for the following analysis. To be specific, the supernatant was used for analyzing ABP degradation, SCFAs production, and non-targeted metabolome, while the precipitation was used for 16S rRNA gene sequencing analysis. The phonel-sulfate method was used to determine ABP degradation.

### 2.4. SCFAs Analysis

The pretreatment method of fermentation supernatant (550 μL) referred to a previous study [18]. The SCFAs level was analyzed using a GC-MS system (Thermo Fisher GCMS-QP2010, Waltham, MA, USA) equipped with an Rtx-5MS column and a flame ionization detector. The detailed protocol was also performed as described previously [19]. 

### 2.5. Microbiome Analysis

The microbial genomic DNA was extracted with a FastDNA SPIN kit for feces (MP Biomedicals). The primers 341F (5′- CCTAYGGGRBGCASCAG -3′) and 806R (5′- GGACTACNNGGGTATCTAAT -3′) were used to amplify the V3-V4 region to obtain 16s data. In addition, species-specific primers *rpsD* (F: 5′- AWCDAGAATHGCMCGTAA -3′, R: 5′- YRTCCCAYTCCAACCA- 3′) and *groEL* (F: 5′- TCCGATTACGAYCGYGAGAAGCT -3′, R: 5′- CSGCYTCGGTSGTCAGGAACAG -3′) were used to determine the species-level relative abundance of *Bacteroides* spp. and *Bifidobacteria* spp. [20,21]. PCR products were sequenced with a Miseq sequencer (Illumina Miseq PE300). Data were analyzed using Qiime2 software (version 1.9.1, Flagstaff, AZ, USA) and the MicrobiomeAnalyst online website. Linear discriminant analysis effect size (LEfSe) was used to distinguish the potentially significant differences between groups. The functional profiles of gut microbes were predicted using PICRUSt.

### 2.6. Untargeted Metabolomics Analysis

The pretreatment method of fermentation supernatant and LC-MS analysis were performed according to a previous study [18]. The raw LC-MS data were converted into visual results using Compound Discoverer 3.3, and then analyzed using Simca 14.1 and online tools, including MetaboAnalyst 5.0 (https://www.metaboanalyst.ca/MetaboAnalyst/ModuleView.xhtml (accessed on 3 January 2022)) and Wekemo Bioincloud (https://bioincloud.tech/task-meta (accessed on 5 January 2022)).

### 2.7. Statistical Analysis

Data were expressed as mean ± standard deviation (SD) and were analyzed using GraphPad Prism 8.3 software. A *p* value < 0.05 was considered statistically significant. An online website (https://hiplot.com.cn/cloud-tool/drawing-tool/list (accessed on 9 January 2022)) was used to analyze the correlation. 

## 3. Results

### 3.1. The Changes in ABP Level, pH, and OD_600_ Values

**Figure 1:**
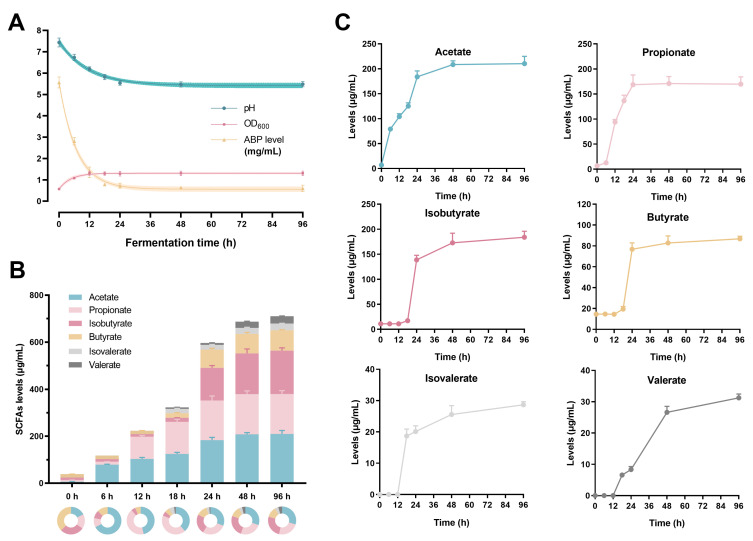
The ABP degradation and SCFAs production during *in vitro* human fecal fermentation. (**A**) ABP level, pH and OD_600_ values at 0, 6, 12, 24, 48 and 96 h; (**B**,**C**) the SCFAs levels.

The ABP content, the values of pH, and the OD_600_ were measured to reflect the polysaccharide consumption, the alterations of the fermentation environment and gut microbial growth. The ABP level reduced sharply from 5.56 to 0.73 mg/mL (more than 85% utilization) within the first 24 h fermentation, and then slowly decreased to approximately 0.60 mg/mL in the following 72 h (Figure 1A). Similarly, the pH value significantly decreased from 7.43 to 5.50 during 0–24 h, and then remained stable (Figure 1A). The initial OD_600_ value was 0.57, which increased dramatically to 1.31 after 12 h fermentation and then remained stable. The results showed that the polysaccharide consumption, the pH value of the fermentation environment, and gut microbial growth basically reached a stable level within 24 h. 

### 3.2. The Production of the SCFAs

The levels of six SCFAs, including acetate, propionate, isobutyrate, butyrate, isovalerate and valerate, were measured during the 96 h fermentation process. As shown in Figure 1B,C, almost all six SCFAs increased with fermentation and reached equilibrium at 24 h, except for valerate. The total SCFAs content increased from 38.98 (0 h) to 596.73 μg/mL (24 h) by more than 15-fold. The levels of the first two SCFAs were highest, followed by isobutyrate and butyrate, and the remaining two had the lowest contents. The results suggested that ABP contributed to increasing SCFAs levels. 

### 3.3. The Diversity of Gut Microbiota

**Figure 2:**
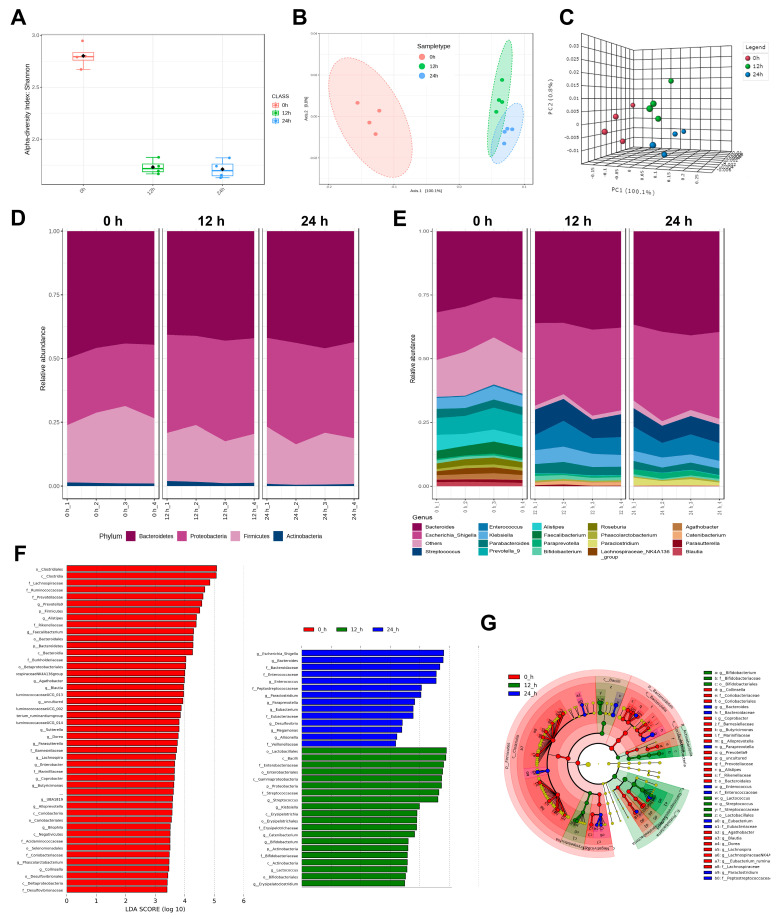
The effects of ABP on the gut microbiota. (**A**) alpha diversity; (**B**,**C**) beta diversity; (**D**,**E**) the composition of gut microbiota at phylum and genus levels, respectively; (**F**,**G**) histograms of LDA scores and circular cladograms, respectively, for statistically significant differences among the 0 h, 12 h and 24 h groups based on the LefSe analysis. p, phylum; c, class; o, order; f, family; g, genus. The diameters of the circles (**G**) are positively related to the relative abundances. Red, green, blue, and yellow circles indicate microorganisms that are significantly enriched in the 0 h, 12 h and 24 h group, or are not significantly affected, respectively.

ABP consumption and SCFAs production were basically stable at 24 h, thus the effects of ABP on gut microbiota were only considered during the first 24 h. Alpha diversity can be reflected by the Shannon indexes, which are usually used to estimate microbial diversity [22]. It decreased at 12 h, possibly due to the great differences in the *in vitro* and in vivo environments, and then remained stable as fermentation progressed (Figure 2A). Zhang et al. also found similar results in the *in vitro* fermentation of heparin [18]. The results of beta diversity showed that the overall structure of the gut microbiota in the 0 h group differed from that of the 12 h and 24 h groups, indicating that the fermentation of ABP significantly altered the structure of the gut microbiota (Figure 2B,C). 

### 3.4. The Composition of Gut Microbiota

**Figure 3:**
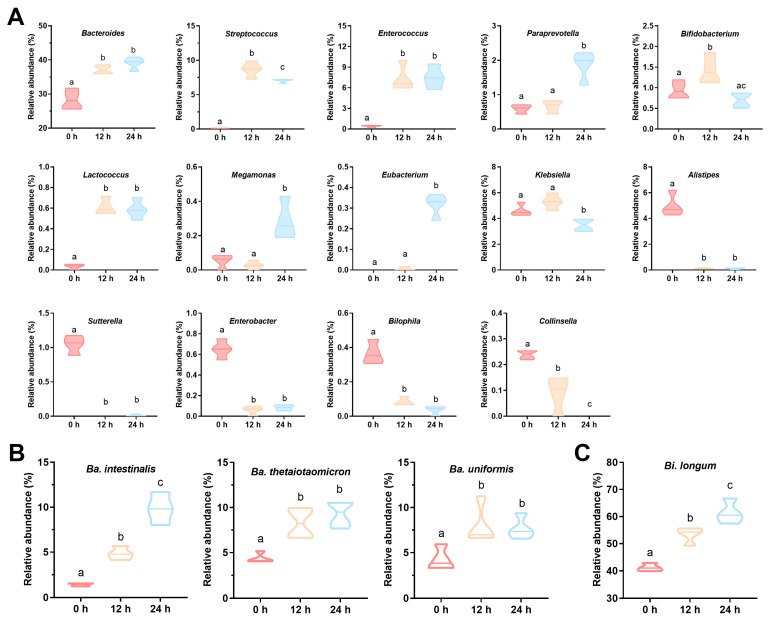
The effects of ABP on the relative abundances of specific gut bacteria at genus (**A**) and species (**B**,**C**) levels. Different letters indicate statistically significant changes among the four groups (*p* < 0.05).

The effects of ABP on the composition of gut microbiota at the phylum and genus levels were analyzed (Figure 2D,E). LEfSe analysis was performed on samples at 0, 12 and 24 h to identify the major changing bacteria (Figure 2F,G). At the phylum level, the relative abundance of Bacteroidetes was the highest (approximately 50%) with no obvious change during 24 h of *in vitro* fermentation; the abundance of Firmicutes was reduced, while Proteobacteria and Fusobacteria were increased (Figure 2D). At the genus level, the main response bacteria for ABP were *Bacteroides*, *Bifidobacterium*, *Streptococcus*, *Eubacterium*, *Paraprevotella*, *Enterococcus*, *Lactococcus*, and *Megamonas*, whose relative abundance significantly increased (*p* < 0.05, Figure 2E–G and Figure 3A). The genera with reduced relative abundance included *Klebsiella*, *Alistipes*, *Sutterella*, *Enterbacter*, *Bilophila*, and *Collinsella* (*p* < 0.05). 

Obviously, the amount of *Bacteroides* was the most abundant and varied significantly. *Bifidobacterium* was an important beneficial bacterium. Therefore, the effect of ABP on these two genera at the species level was further determined. A total of 16 *Bacteroides* and six *Bifidobacterium* species were detected. However, the relative abundances of some species, such as *Ba. acidifaciens*, *Ba. eggerthii*, *Ba. finegoldii*, *Bi. animalis*, and *Bi. breve*, were very low, while the relative abundances of some other species, such as *Ba. vulgatus*, *Ba. ovatus*, *Bi. adolescentis*, and *Bi. ruminantium*, decreased or remained constant during ABP fermentation. Thus, the species significantly enriched during 24 h of ABP fermentation were *Ba. thetaiotaomicron*, *Ba. intestinalis*, *Ba. uniformis*, and *Bi. longum* (*p* < 0.05, Figure 3B,C). 

### 3.5. Functional Profile of the Gut Microbiota

**Figure 4:**
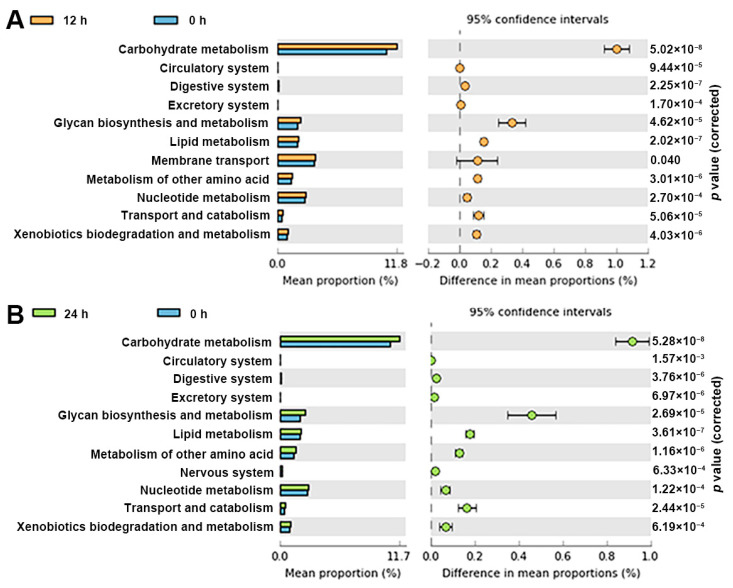
Effects of ABP on the functional profile of the gut microbiota. (**A**) The enriched KEGG pathways in 12 h fermentation at level 2, (**B**) the enriched KEGG pathways in 24 h fermentation at level 2.

The results of PICRUSt showed that 43 (level 2) and 292 (level 3) Kyoto Encyclopedia of Genes and Genomes (KEGG) pathways were identified. ABP markedly enriched 11 KEGG pathways in level 2 after 12 or 24 h of *in vitro* fermentation (*p* < 0.05), in which the top five pathways were carbohydrate metabolism, glycan biosynthesis and metabolism, lipid, amino acid and nucleotide metabolism (Figure 4). It is worth mentioning that the nerves system pathway was enriched only in the 24 h group, indicating that ABP has the potential to alleviate nervous injury by regulating gut microbiota [17].

### 3.6. The Effects of ABP on Metabolic Changes

**Figure 5:**
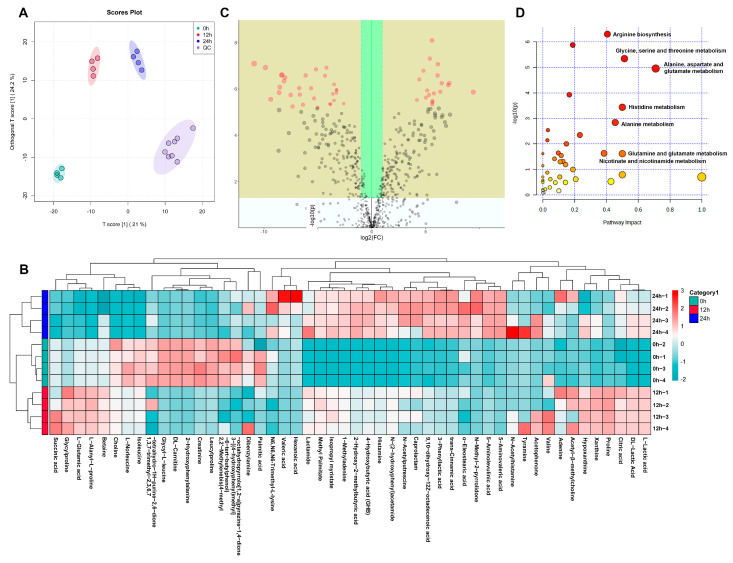
ABP-induced metabolic changes during *in vitro* fermentation. (**A**) OPLS-DA score plot from the metabolome datasets. (**B**) The cluster heatmap analysis of different metabolites. (**C**) The different metabolites between 0 and 24 h of fermentation. (**D**) Pathway analysis plots between 0 and 24 h of fermentation.

A total of 390 metabolites was obtained after screening and merging the data in positive and negative ion modes. The results of the OPLS-DA analysis showed that the quality control (QC) group clustered together, indicating stable meter operation and slight data errors, while the 0 h, 12 h and 24 h groups showed a clear separation, indicating that the metabolites in each group were significantly different (Figure 5A). In addition, a clustering and heat map analysis of the metabolites was performed. Figure 5B showed the clustering of the top 50 metabolites in abundance and grouping. A number of SCFAs, such as succinic acid, DL-Lactic acid, 2-Hydroxy-2-methylbutyric acid, 4-Hydroxybutyric acid, 3-phenyllactic acid, 5-Aminolevulinic acid, and 5-Aminovaleric acid, were clustered in 12 h and 24 h groups.

VIP value was obtained by OPLS-DA analysis, and the magnitude of change can be measured by the fold change (FC) of metabolites. As shown in Figure 5C, the yellow areas are metabolites that satisfy *p* < 0.5 and FC > 2 or FC < 0.5, indicating that these metabolites are significantly different between the 0 h and 24 h groups. Ultimately, 18 metabolites, such as DL-Tryptophan, L-Glutamine, pipecolic acid, pyruvic acid, gamma-aminobutyric acid (GABA), indole-3-acetic acid, indole-3-lactic acid, 2-hydroxyvaleric acid, linoleic acid, 2-aminonicotinic acid, nicotinamide, nicotinamide adenine dinucleotide (NAD^+^), nicotinic acid mononucleotide, hypoxanthine, and acetylcholine, were identified and screened out as the potential biomarkers (Table 1). These metabolites were involved in various pathways, including amino acid, carbohydrate, lipid, nucleotide, nicotinate, and nicotinamide metabolism pathways. In addition, the top three increased metabolites in the 24 h group were nicotinic acid mononucleotide, indole-3-lactic acid, and NAD^+^, which increased 70.34, 56.10, and 15.36 times, respectively (*p* < 0.05). 

Pathway analysis was performed for different metabolites between 0 h and 24 h groups, which can indicate the significantly affected metabolic routes during the *in vitro* fermentation of ABP (Figure 5D). The results showed that the significantly affected pathways were amino acid synthesis and metabolism, including arginine biosynthesis, glycine, serine, threonine, alanine, aspartate, glutamine, glutamate, histidine, and proline metabolism, and nicotinate and nicotinamide metabolism (*p* < 0.05). 

### 3.7. Spearman Correlation Analysis

**Figure 6:**
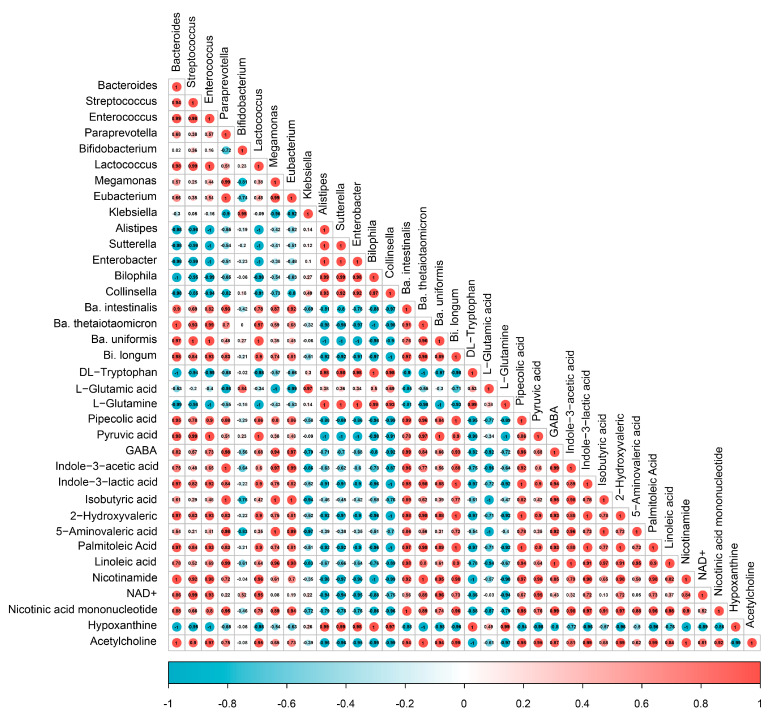
The Spearman correlation analysis between specific gut microbiota and metabolites.

A Spearman correlation analysis was used to explore the correlation between specific gut microbiota and metabolites (Figure 6). Specifically, the relative abundances of *Bacteroides*, *Streptococcus*, *Enterococcus*, and *Lactococcus* were strongly negatively correlated with the tryptophan, L-glutamine, and hypoxanthine, while positively correlated with pyruvic acid, indole-3-lactic acid, 2-hydroxyvaleric acid, palmitoleic acid, nicotinamide, NAD ^+^, and acetylcholine (|r| > 0.80). The *Paraprevotella*, *Megamonas*, and *Eubacterium* were strongly negatively correlated with the L-glutamic acid, while positively correlated with pipecolic acid, GABA, indole-3-acetic acid, isobutyric acid, 5-aminovaleric acid, linoleic acid, and nicotinic acid mononucleotide (|r| > 0.80). At the species level, the *Ba. thetaiotaomicron*, *Ba. intestinalis*, *Ba. uniformis*, and *Bi. longum* were strongly negatively associated with tryptophan, L-glutamic acid, and hypoxanthine, while positively correlated with pipecolic acid, indole-3-acetic acid, 2-hydroxyvaleric acid, palmitoleic acid, nicotinamide, and acetylcholine (|r| > 0.80). The results indicated that ABP enriched these beneficial metabolites by regulating the relative abundances of *Bacteroides*, *Streptococcus*, *Enterococcus*, *Lactococcus*, *Paraprevotella*, *Megamonas*, and *Eubacterium*.

## 4. Discussion

The gut microbiota is a dynamic microbial community and is considered a super organ that can regulate the host metabolism [23]. The diversity and composition of gut microbiota could be affected by *A. bispours* [10]. The ABP, as an important active compound in *A. bispours*, is a mixture of mannogalactan (55.8%), (1→4)-(1→6)-α-d-glucan and (1→6)-β-d-glucans [24]. The monosaccharide composition of ABP is mainly glucose (more than 95%), with some minor proportions of galactose and xylose [25]. Its immunostimulatory and anti-inflammatory activities have been reported [11,12,13], but its interaction with gut microbiota is unclear.

*In vitro* batch fermentation is the simplest methodology to simulate colonic fermentation [16]. It has been widely used to study the interaction of gut microbiota with specific foods or food components (such as polysaccharides) [18]. Although it cannot accurately reflect the complex interactions that occur in the intestine, it also has many advantages. For example, (i) it can help elucidate the metabolic routes involved and the intermediate metabolites appearing by exposing gut microbiota to certain compounds; (ii) this could, in turn, provide clues on how to promote microbial metabolism toward specific goals, such as promoting the growth of specific beneficial bacteria or the production of particular beneficial metabolites; (iii) it is much less time- and cost-consuming than in animal or human studies [16]. Therefore, it was decided to test the interaction of ABP with gut microbiota in our study. Due to the great interindividual variability between different individuals, fresh fecal samples were collected and pooled from five volunteers. Pooling will ensure that keystone microbes are not missing, which could result in compounds not being metabolized [16]. 

*Bacteroides* spp. is a core dominant genus in the gut; therefore, it has a global effect on both the host and the gut microbiota. For example, the administration of *Ba. thetaiotaomicron* could alleviate diet-induced metabolic disorders and mitigate obesity of the host [26]. In the microbial community, *Bacteroides* spp. also play a vital role because, as a nutrition provider, it can support other microorganisms to colonize and grow in the intestine [27]. In addition, *Bacteroides* has plenty of outer surface glycoside hydrolases, which degrade a wide range of polysaccharides [28]. Its versatility may help to explain their high abundance in the colon [29]. For example, *Ba. thetaiotaomicron* can break down fructose polymers to fructo-oligosaccharides, then monomeric fructose [30]. Interestingly, different strains consumed the individual polysaccharide in different orders and speeds as their different preferences, facilitating their coexistence in microbial communities [31]. In this study, *Ba. thetaiotaomicron*, *Ba. intestinalis*, and *Ba. uniformis*, rather than *Ba. vulgatus*, could rapidly utilize ABP to produce various monosaccharides and metabolites. 

*Bifidobacterium* spp. plays an important role in human health and metabolism. Its abundance in patients suffering from diarrhea, allergy, necrotizing enterocolitis, and obesity is significantly decreased [32]. For example, it can covert aromatic amino acids (tryptophan, phenylalanine and tyrosine) to aromatic lactic acids (indolelactic acid) via aromatic lactate dehydrogenase (ALDH) in the infant gut, which was correlated with the activation of aryl hydrocarbon receptor (AhR), thereby maintaining intestinal homeostasis and immune response [33]. *Bifidobacterium* also evolved the capacity to use complex carbohydrates to thrive in the competitive niche [32]. The relative abundance of *Bi. longum* increased gradually within 24 h of *in vitro* fermentation, indicating that it could well utilize ABP. Cordeiro et al. explored the critical biochemical steps involved in high-mannose N-glycan utilization by *Bi. longum*, which involved its functionally distinct glycoside hydrolase 38 [34]. Moreover, various beneficial functions of *Bi. longum*, including anti-aging [35,36] and the alleviation of chronic constipation [37] and depression [38], have attracted increasing attention. 

*Streptococcus* spp. could ferment various polysaccharides to produce lactic acid, and thus its abundance significantly increased when consuming carbohydrate-rich foods, such as wheat flour [39]. It was related to healthier eating behavior and was enriched in a ‘healthy’ microbiota [40], which exerted potential benefits for the host metabolism, such as lower systolic blood pressure [41], while higher dietary fiber or polysaccharides intake was associated with less abundant *Collinsella* in overweight adults [41]. It was correlated with unhealthier eating traits, such as a high-fat–low-fiber diet, and had a pro-inflammatory potential [41,42,43], which was related to higher body fat mass and higher blood glucose [44]. Interestingly, there was a potential antagonistic effect between *Alistipes* and *Streptococcus*. The increase of *Streptococcus* may cause a decline in *Alistipes* [45,46]. *Eubacterium* spp., a genus increased by ABP, was butyrate-producing microbes and was considered beneficial to human health in the same manner as *Bifidobacterium* [47]. It played an important role in energy homeostasis, colonic motility and immunomodulation in the gut [48]. Moreover, *Eubacterium* spp. also performed bile acid and cholesterol conversion in the gut, thereby maintaining their homeostasis [48]. The relative abundances of *Paraprevotella* spp. and *Bilophila* spp. were increased and reduced, respectively, after ABP *in vitro* fermentation. These two genera were associated with Parkinson’s disease (PD) [49]. The relative abundances of *Paraprevotella* showed a decrease in female PD patients [49]. 

SCFAs are a class of widely studied microbial metabolites. The function of the gut microbial is commonly measured by the production of SCFAs (mainly acetate, propionate and butyrate), which are the main microbial fermentation products and are considered beneficial to human health [50,51]. For example, acetate can activate G protein-coupled receptors that boost microbiota and thus regulate adipose–insulin signaling transduction [6]; butyrate can maintain gut barrier integrity and exert immune-modulating, anti-inflammatory and anti-carcinogenic effects [52]; propionate is considered to induce satiety and improve glucose metabolism [53,54]. 

The precursors of GABA, an important neurotransmitter, were glutamate, arginine, putrescine and ornithine. Strandwitz et al. explored the potential GABA production of the gut microbiota [55]. The results showed that *Bacteroides* spp. can activate the expression of GABA-producing pathways and thereby produce large quantities of GABA. The relative abundance of *Bacteroides* was negatively associated with depression, mediated by GABA. *Bifidobacterium* spp. Could also produce GABA via decarboxylation of glutamate at a low pH [56,57]. It is worth noting that GABA production by *Bacteroides* was not only observed at a low pH (≤5.5), but also at a physiologically relevant pH in the human intestine (pH 5.7–7.4) [55]. Pipecolic acid is an intermediate metabolite of L-lysine and could activate GABA receptors [58,59]. The levels of GABA and pipecolic acid decreased in the serum of mice with colitis and depressive-like behaviors, which could be mitigated by pipecolic acid supplementation [60]. 

In addition, NAD^+^, a coenzyme for redox reactions and a central metabolic regulator, played a vital role in maintaining mitochondrial function and host health [61]. NAD^+^ and its enhancers, such as nicotinamide [62], can activate sirtuin 1, promote autophagy, and attenuate oxidative stress and inflammation in aged mice [63,64], which were the most promising interventions to attenuate aging [65,66]. Moreover, L-pipecolic acid could significantly mitigate depressive behaviors in mice with colitis. The anti-aging and ameliorative behavioral deficits of ABP were demonstrated in a previous study [17], which may be due to the increase in these three metabolites (GABA, NAD^+^ and nicotinamide).

Indoles, as a beneficial product of tryptophan metabolism by gut microbiota, play important roles in innate immunity, the suppression of inflammation and the removal of free radicals [67]. Indole-3-lactic acid can be produced by gut bifidobacterial species, such as *Bi. longum* [68,69]. Our results also showed that the indole-3-lactic acid level was significantly positively correlated with the relative abundance of *Bi. longum.* Indole-3-lactic acid exerted its anti-inflammatory effect by interaction with the AhR and decrease of the inflammatory cytokine IL-8 [69]. Henrick et al. found that *Bi. infantis*-derived indole-3-lactic acid upregulated immunoregulatory galectin-1 in T cells during polarization, providing a functional link between beneficial bacteria and immunoregulation in infants [70].

## 5. Conclusions

In conclusion, this study demonstrated that, during the *in vitro* fermentation of ABP, the ABP consumption, the pH of the fermentation environment, gut microbial growth and the production of SCFA basically reached a stable level within the first 24 h. Moreover, the main degrading bacteria for ABP were *Bacteroides*, *Streptococcus*, *Paraprevotella*, and *Bifidobacterium*, whose relative abundances increased, while the abundances of *Klebsiella*, *Alistipes*, *Sutterella*, *Enterbacter*, *Bilophila*, and *Collinsella* decreased. It is worth mentioning that ABP can enrich the species of *Ba. thetaiotaomicron*, *Ba. intestinalis*, *Ba. uniformis*, and *Bi. longum*. The changes in gut microbiota led to more than 10-fold increases in the relative amounts of GABA, nicotinamide and NAD^+^ at 24 h of ABP fermentation. The change in the proportions of these metabolites, which directly or indirectly promote autophagy and attenuate oxidative stress and inflammation, plays an important role in slowing aging and preventing neurodegenerative disorders. Therefore, ABP has the potential to be used as a potential prebiotic or dietary supplement to delay aging by regulating the gut microbiota and its metabolites.

However, the study is limited by its use of *in vitro* batch fermentation, which does not accurately reflect the complex interactions that occur in the human gut. Therefore, further research is needed to fully understand its impact and validate the results in animal models or the human population in the future.

## Figures and Tables

**Table 1:** The significantly changed metabolites during *in vitro* fermentation of ABP.

No.	*m*/*z*	Compound Name	Chemical Formula	Fold Change	
				12 h	24 h
Amino acid metabolism					
1	203.08306	DL-Tryptophan	C_11_ H_12_ N_2_ O_2_	0.26	0.09
2	148.0594	L-Glutamic acid	C_5_ H_9_ N O_4_	1.12	0.33
3	147.07589	L-Glutamine	C_5_ H_10_ N_2_ O_3_	0.35	0.34
4	130.08623	Pipecolic acid	C_4_ H_7_ N O_4_	2.16	3.2
5	104.07035	GABA	C_3_ H_4_ O_3_	4.64	14.43
6	146.11764	Acetylcholine	C_5_ H_4_ N_4_ O	5.36	7.03
7	174.05607	Indole-3-acetic acid	C_4_ H_9_ N O_2_	1.17	2.05
8	204.06669	Indole-3-lactic acid	C_10_ H_9_ N O_2_	33.46	56.10
Carbohydrate metabolism					
9	87.04528	Isobutyric acid	C_11_ H_11_ N O_3_	0.45	10.63
10	117.05603	2-Hydroxyvaleric acid	C_4_ H_8_ O_2_	4.29	6.51
11	118.08565	5-Aminovaleric acid	C_5_ H_10_ O_3_	0.58	3.6
12	87.00884	Pyruvic acid	C_6_ H_11_ N O_2_	2.88	2.8
Lipid metabolism					
13	313.2391	Palmitoleic acid	C_5_ H_11_ N O_2_	2.1	2.82
14	279.2337	Linoleic acid	C_16_ H_30_ O_2_	2.64	8.75
Nicotinate and nicotinamide metabolism					
15	123.05496	Nicotinamide	C_18_ H_32_ O_2_	8.88	11.34
16	664.11414	NAD^+^	C_6_ H_6_ N_2_ O	10.38	15.36
17	336.04694	Nicotinic acid mononucleotide	C_21_ H_27_ N_7_ O_14_ P_2_	26.83	70.34
Nucleotide metabolism					
18	135.03142	Hypoxanthine	C_11_ H_14_ N O_9_ P	0.35	0.24

Fold change: The fold change is compared to 0 h. GABA, gamma-aminobutyric acid; NAD^+^, nicotinamide adenine dinucleotide.

## Data Availability

Data is contained within the article.
